# Analysis of plant gene family heat shock protein 100 (HSP100) and its orthologs in Eukarya reveals sites of divergent evolution and insights into endosymbiotic origins of chloroplasts

**DOI:** 10.1080/15592324.2025.2532008

**Published:** 2025-07-20

**Authors:** G. Gallas

**Affiliations:** Department of Biology, San Diego State University, San Diego, CA, USA

**Keywords:** Heat shock protein 100, caseinolytic protease B, molecular chaperones, ATPases associated with various cellular activities

## Abstract

Heat shock protein 100s (HSP100s) is present in bacteria, basal eukaryotes, plants, and fungi, but not in metazoans. While this protein family has been extensively studied in plants in terms of structure and functionality, this research addresses a gap in knowledge about their evolution and origins in eukaryotic groups. Protein sequences were aligned, and phylogenetic relationships were inferred. Amino acid substitution model testing was performed using ProtTest. These results recovered six homologues of plant HSP100s, consistent with previous literature, and also two major lineages of fungal HSP100s. At least one of these two fungal homologues of HSP100 are present in all basal eukaryotic groups studied, indicating their origins at the base of the eukaryotic phylogeny. HSP100 homologs are also present in choanoflagellates (earliest diverging animal lineage), indicating they were later secondarily lost in all other metazoans. Plants have additional 3 copies of HSP100s, obtained from a cyanobacterial ancestor that was the endosymbiotic precursor to the chloroplast. Finally, several groups within the Heterokonts are of particular interest due to serial plastid endosymbiosis, resulting in the presence of some but not all HSP100 proteins in these lineages. This study reveals the complex evolutionary history of this important molecular chaperone protein family.

## Introduction

The ClpB/heat shock protein (HSP) 100 proteins are members of the protein superfamily of AAA+ ATPases associated with various cellular activities.^1^ These proteins are found in both prokaryotes and eukaryotes and function as molecular chaperones that involve the ATP-dependent unfolding, disassociation, and re-folding of nucleic acids and proteins.^2^ ClpB/HSP100 chaperone proteins, in conjunction with the HSP70/DnaK chaperone systems, confer thermotolerance by mediating the retrieval and reactivation of stress-denatured proteins.^3^ In bacteria, proteins of this type were first recognized as caseinolytic proteases (Clp) that function as ATP-dependent protease/unfoldases in *Escherichia coli*.^4^ In the yeast *Saccharomyces cerevisiae*, this protein subfamily is known as HSP104, and they cooperate with the fungal chaperone system Hsp70 and mitochondrial Hsp78 to refold heat-denatured proteins and are critical in acquired thermotolerance.^5,6^ In the plant *Arabidopsis thaliana*, the HSP100/Clp protein family are notable heat-stress induced proteins of around 100-kDa, and homologues have been characterized across many plant species.^7^ Protein homologues of ClpB/HSP100 are widely dispersed across the tree of life, suggesting an ancient and highly conserved function, and are found in bacteria, basal eukaryotes, plants, and fungi.^8,9^ Remarkably, homologues of this protein family are conspicuously absent in metazoans.^10^

Proteins in the ATP-dependent caseinolytic protein family were first discovered in bacteria and yeast as important constitutively expressed proteins that function as stress signals and are required for tolerance to heat stress.^6,11,12^ In plants, homologues of these proteins were discovered in rice that had molecular weights of around 100 kDa.^7,13^ Additional members of the Clp family of proteins were discovered in many other plant species and were found to be induced by heat stress and in fact, necessary for the acquisition of heat tolerance in *Arabidopsis thaliana*.^14,15^ Proteins in this family prevent the accumulation of unfolded, partially folded, or misfolded proteins that can aggregate in the cell and if unchecked, lead to cell death.^16^ Molecular chaperones including but not limited to ClpB/Hsp100 proteins perform these functions in various ways: by binding to and therefore shielding hydrophobic regions of partially folded or unfolded proteins, by unfolding and then degrading partially folded or misfolded proteins or by releasing improperly bound polypeptides and rebinding stably to protein complexes.^17^ Protein disaggregation relies on the collaboration between ClpB/Hsp104 with a related Hsp70 chaperone system.^16^ Bacterial ClpB and its homologues in yeast and plants use ATP to refold proteins that have been improperly folded due to stressful conditions (such as heat stress) in a nondestructive manner, rather than by degrading these proteins.^18^ This allows the resolubilization of protein aggregates when temperatures return to normal.^19^ However, in plants, Clps function not only to resolubilize misfolded proteins in a nondestructive manner but also have the unique ability to remove harmful aggregated proteins following stress.^20^ The evolution of ClpB genes in plants has become more complex due to the sessile nature of plants, which cannot move away from sources of stress, unlike bacteria, yeast, or even animals, which lack ClpBs altogether.^20^

**Figure 1.**
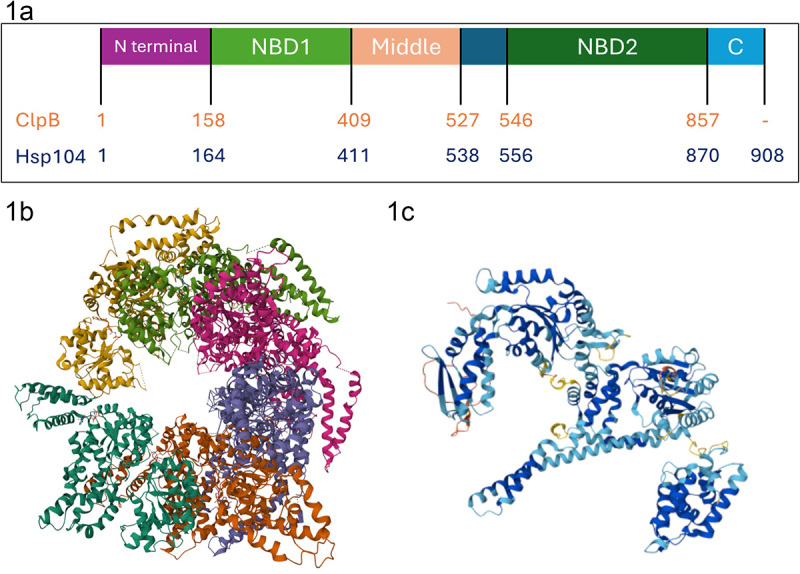
A) structure of HSP100. HSP100/ClpB is represented here as a monomer. ClpB (bacterial origin) residue numbers shown in orange (top), and Hsp104 (from yeast) residue numbers in blue (bottom). Domain organization shown here. NBD1 represents the nucleotide binding domain 1, in green. MD represents the middle domain, shown in peach. NBD2 represents the nucleotide binding domain 2, shown in dark green. C represents the C-terminal domain shown in cyan, only Hsp104 has the C-terminal extension. (Figure reconstructed with permission from M.E. Desantis & J. Shorter (2012)^32^. b) CryoEM reconstruction of Hsp104 Hexamer. 3D top-down view of the ring-shaped hexamer of Hsp104. Note the axial channel through which aggregated proteins are disaggregated. Each monomer of Hsp104 shown in different color. Figure used with permission from D. R. Southworth et al. (2016)^33^. c) Alphafold prediction of *Arabidopsis thaliana* ClpB1 structure. Predicted structure of monomeric *A. thaliana* ClpB1 protein (Uniprot accession# P42730) using AlphaFold. AlphaFold prediction used with permission Varadi et al. (2024) & J. Jumper et al. (2021)^34,35^.

HSP100/ClpB are protein disagregases that function as ring-shaped hexamers.^4^ The HSP100/ClpB proteins are part of a larger protein family of AAA+ ATPases. They have notable chaperone functions, assisting in regulation of protein complexes, unfolding of proteins for presentation to proteases using ATP, refolding denatured proteins, and they interact with and disaggregate misfolded proteins.^8,21^ It is generally understood that the resolubilization of aggregated proteins is facilitated by movement through the central channel between the hexameric units.^22,23^ A 3D top-down representation of Hsp104 can be seen in Figure 1B.^24^ Each HSP100/ClpB protein is comprised of two signature AAA+ family nucleotide-binding domains, a ‘middle’ or ‘linker’ domain located between these domains, and N and C-terminal regions of the protein which assist in hexamerization.^9,23,25^ Figure 1 depicts a monomeric representation of ClpB (top) and Hsp104 (bottom) domain structures.^23^ The monomeric folded protein structure predicted by AlphaFold is shown in Figure 1C.^26^ *In vivo*, the protein functions as a hexameric structure (see Figure 1B) and this complex is crucial for the chaperone activity of the protein.^22^ Loss of either N or C-terminal domains of the protein results in a loss of chaperone function.^22^ Mutational analysis of the nucleotide-binding domains (NBDs) indicate that these domains are essential in proper formation of the protein hexamer.^27,28^ The NBDs are well conserved in bacteria, fungi, plants and the mitochondria of eukaryotes.^4,29^ In some homologues, such as Hsp104, the NBD1 contributes the majority of the ATPase activity, in other homologues, such as ClpB, both NBDs are required.^11,30^ The coil-coiled middle domain, that links the two nucleotide binding domains, is essential for thermotolerance, alters ATPase function, facilitates communication between the NBDs, and stabilizes the hexamer structure.^6,23,28,31^

While many studies have examined the function and expression of HSP100s^8,15,31,36–38^ to date, there have been few studies examining the phylogenetics of this protein in the context of functional divergence.^8^ One such study by Lee et al. (2007) examined the amino acid sequences of HSP100 and its homologues using neighbor-joining, maximum likelihood, and Bayesian frameworks.^8^ The goal of this study was to explore the function of these proteins in terms of their intracellular localization, expression patterns, and behavior of mutants deficient in these proteins in an evolutionary context,^8^ rather than to infer phylogenetic relationships between large groups of eukaryotes. In addition, this study was limited to plants. Another study that critically reviewed the ClpB/Hsp100 proteins in plants and their role in tolerance to heat stress by Mishra and Grover (2015) also examined the diversity of Clp proteins and their phylogenetic relationships within plants.^20^ While this study provides an excellent review of Clp proteins, their functions, distributions, their protein structure, and their expression and function, it is focused only on Clp proteins in plants.

Here, we have examined complete genomes of organisms that are representative of key Eukaryotic lineages for the presence of HSP100/ClpB homologs and have used phylogenetic analysis to examine the evolutionary history of this protein family. The amino acid sequences of these homologues have been placed phylogenetically on the tree of life and gains and losses of these proteins were noted. In addition, the domains of these proteins have been analyzed to identify amino acid residues with distinct conservation patterns. These analyses characterize the differences of these proteins across the tree of life and highlight where losses and expansions occurred in the evolutionary history of Eukaryotes. Our analysis confirms and strengthens previous research by Erives & Fassler that concluded that the ClpB chaperones HSP104 and HSP78 were present in an ancestral eukaryote and retained in fungi and choanoflagellates but were secondarily lost in the animal lineage.^10^

Six homologues of HSP100/Clp have been identified from the complete genome of *A. thaliana*: HSP101, two ClpCs, one ClpD, and two ClpBs – one localized to the mitochondria and one localized to the chloroplast.^9^ In some treatments including many involving plant Clps, these proteins are semantically categorized as ‘three’ copies of ClpB: one around 100 kDa that is localized to the cytoplasm, and two additional copies localized to organelles:, one localized to plastids, and one localized to mitochondria.^20^ Due to the homology between cyanobacterial and plant ClpBs, it seems likely that the endosymbiotic event that produced the chloroplast from a cyanobacterial endosymbiont is also the evolutionary origin of the plant HSP101 and ClpB homologues.^9^ However, the origins of this protein family in fungi and other eukaryotes is as yet unknown: whether they also have endosymbiotic origins, originated in horizontal gene transfer, or had an independent evolutionary origin. The lack of ClpB/HSP100 homologs in metazoans is mysterious and warrants further phylogenetic investigation. Here, we have examined complete genomes of organisms that are representative of key Eukaryotic lineages for the presence of HSP100/ClpB homologs and have used phylogenetic analysis to determine the evolutionary history of this protein family. This analysis allows us to discover information about the origins of genes within this family and identify lineages where gains or losses have occurred. Phylogenetic analysis characterizes the differences of these proteins across the tree of life and the domains of these proteins have been analyzed to identify amino acid residues with distinct conservation patterns. Here, we present the largest to-date phylogenetic analysis of the ClpB/HSP100 protein family that allows us to infer phylogenetic relationships of these proteins between large groups of Eukaryotes and reveals interesting patterns of gains and losses that suggest an interesting history of evolutionary divergence of these proteins.

## Methods

### Data source

Protein sequences were obtained via Basic Local Alignment Searches (BLASTP) of non-redundant protein sequences on the National Center for Biotechnology Information (NCBI) Genbank database site^39^ and from the Department of Energy’s Joint Genome Institute (JGI) BLASTP database.^40^ Specific resources were used for taxa with individual databases, such as Phytozome for plants,^41^ and Mycocosm for fungal genomes.^42^ Additional genomes that were not hosted on the above sites but were also mined for this project included: the Broad Institute’s Origins of Multicellularity Database,^43^ the Norway Spruce genome^44^ to represent the gymnosperms, and the *Amborella* genome^45^ to represent the earliest diverging Angiosperms. These proteins have not been observed in Archaea.^7^ The protein sequences that were used for the BLAST search for the plant genomes were the six *Arabidopsis thaliana* homologues for HSP100 (At1g74310, At5g15450, At2g25140, At3g48870, At5g51070, At5g50920) which were described in detail in Lee et al. 2007.^8^ These sequences were gathered from The Arabidopsis Information Resource (TAIR) and used in BLASTP.^46^ To search for the sequences from the fungal genomes, the two homologues from the *Saccaromyces cerevisiae* were used for the BLASTP search. To search for the sequences in bacterial genomes, the two sequences from *Escherichia coli* were used. The protein sequences that were included had expect values of 0.0 or smaller than 1*e^−50^, indicating a high level of sequence alignment and probable homology.^47^

### Taxon sampling

The taxa were selected on the basis of: 1) phylogenetic coverage, and 2) complete genome availablity. A representative taxon was sampled from the available complete genomes from all of the major plant, basal eukaryote, and fungal lineages. The bacterial taxa were sampled from all major bacterial lineages, with more emphasis on cyanobacteria and other lineages that have close endosymbiotic associations with plants. A representative species was chosen from each large taxonomic group of basal eukaryotes, fungi, and Viridiplantae.

Of particular note, there have been no reported identifications of HSP100/ClpB protein homologues in the domain Archaea.^48^ Studies of microbes in extreme environments have suggested that chaperone proteins such as DnaK and ClpB may be important for stress responses in extremophiles and counterparts of these chaperones have been predicted from metagenomic studies of hypersaline lakes.^49^ A BLASTp search of representative complete genomes of Archaea was performed using the *Escherechia coli* and the *A. thaliana* ClpB amino acid sequences. This search included at least one species from each major group of the Archaea: Crenarchaeota, Euryarchaeota, Korarchaeota, Nanoarchaeota, and Thaumarchaeota. The bacterial ClpB search yielded only two results with expect values much higher than our threshold and sequence identities of around 40% from a species of Euryarchaeota. The plant ClpB search also yielded two results with expect values much higher than our threshold and sequence identities of around 30% from a species of Korarchaeota and one species of Crenarchaeota. It is therefore suggested that these proteins may be other distantly related AAA+ family ATPases, and they are classified as such.^50^ These proteins may represent evolutionary precursors to HSP100s/ClpB proteins that may have functional importance in the evolution of eukaryotes.^49^

### Protein sequence alignment and model selection

Protein sequences were aligned in Mesquite^51^ using the OPAL software using default parameters.^52^ To verify this alignment, the protein sequences were also aligned using MAFFT,^53^ TCOFFEE,^54^ and CLUSTALW^55^ implemented in the online Cyberinfrastructure for Phylogenetic Research (CIPRES) Science Gateway using default parameters.^56^ Final sequence alignments of the protein sequences were done using PROMALS3D.^57^ The final alignment was verified and adjusted manually using the program BioEdit.^58^ Ambiguous, poorly aligned regions, transit sequences, and regions unique to one or a few sequences were removed to reduce alignment error which could result in less accurate downstream analyses.^59^ The final alignment was compared to the automated alignment tool for large-scale phylogenetic analyses, trimAI, utilized in CIPRES.^60^ Alignments were re-iterated several times to ensure accuracy, and no significant differences were found between the alignment methods.

Ninety-four OPAL-aligned protein sequences were loaded into the program ProtTest,^35,61,62^ which selects a model of protein evolution that best fits a given alignment using empirical protein substitution matrices, relative rates of amino acid replacements, and rate heterogeneity between amino acid sites. This program selects the best model of protein evolution based on Akaike Information Criterion (AIC) framework, Bayesian Information Criterion (BIC) framework, and Maximum likelihood framework (produces log likelihood (−lnL) scores).^61^ ProtTest selected LG+I+G+F as the best model of protein evolution for our alignment: Lee and Gascuel amino acid replacement matrix (LG) plus invariant sites (I) plus Gamma (G) plus amino acid frequency heterogeneity (F)^63,64^. The confidence of this model was: 227,507.75 as measured by the Akaike Information Criterion (AIC) framework, 228,615.18 as measured by the Bayesian Information Criterion (BIC) framework, and the log likelihood (−lnL) score was 113,547.87 found by Maximum likelihood analysis.^61^

### AlphaFold structure prediction

AlphaFold uses a neural network to accurately predict protein structures to near experimental accuracy.^26^ AlphaFold was utilized to predict the monomeric protein folding structure of *Arabidopsis thaliana* ClpB1 (Uniprot# P42730).

### Phylogenetic methods and confidence assessment

Maximum-likelihood (ML) analyses were performed using RAxML 8.0.9^65,66^ implemented on the CIPRES Science Gateway.^56^ For the protein analysis, RAxML has more flexibility in its choices of amino acid substitution matrices, so the preferred matrix as determined by ProtTest was used. The model selected by ProtTest was the LG amino acid replacement matrix as described by Le and Gascuel in 2008,^63^ with invariant sites (I), gamma (G), and heterogeneity between frequencies of different amino acids (F). To reduce computational burden, RAxML on CIPRES allows either a gamma (Protein GAMMA) distribution to be estimated, or estimates a subset of gamma categories to estimate the parameter α (Protein CAT), which approximates gamma for larger datasets.^67^ Protein CAT was used to analyze this dataset. Invariant sites, I, is not implemented in this version of RAxML. Heterogeneity between the frequencies of amino acids in a dataset, the parameter F, is implemented in that it allows empirical frequencies to be used rather than imposing equal frequencies.^68^ The phylogenetic trees were rooted using the ClpA subfamily of *Escherichia coli* and *Salmonella enterica* ^8^ as the bacterial outgroups. These two sequences came out as monophyletic in all analyses and trees were rooted ad-hoc using these sequences.

To assess support of the maximum likelihood trees, bootstrapping was performed using the RAxML CIPRES interface.^56,64,65^ This was a rapid bootstrap analysis,^68^ and bootstrapping was conducted for 5000 bootstrap replicates (the maximum allowed), and the optimal tree was reported with bootstrap values. Trees were edited in FigTree v1.4.^69^ Clades that had bootstrap support values of 90% or better were considered to be well supported. Phylogenetic trees produced by RAxML on CIPRES were compared to phylogenetic trees produced by IQ-TREE utilized on CIPRES.^70^ The IQ-Tree reveals nearly the same topology as the RAxML phylogeny, highlighting the reproducibility of our analysis and supporting its accuracy.

### Analysis of sites undergoing divergent evolution using DIVERGE

Using the phylogeny-based program, DIVERGE 3.0, the evolution of the HSP100/ClpB gene family was further examined.^71,72^ Using the multiple sequence alignment, DIVERGE evaluates patterns of rate change at amino acid sites to discern functional constraint patterns within a gene family.^71^ DIVERGE employs statistical tools to identify sites that are likely to have undergone divergent evolution after a gene duplication event.^71^ These sites are likely to be important for functional divergence in the evolution of the protein family.^71^ Aligned amino acid sequences were loaded into the program, and clusters were defined by importing phylogenetic trees made using the above methods. Parameters were then calculated using the program, with bootstrapping where appropriate. Sites that underwent significant divergence were identified using cutoff values as specified in Tables 1–3. Further investigation can be facilitated by the identification of these sites into functional significance, as we have matched these sites from our alignment up with *Arabidopsis thaliana* and *Saccharomyces* reference sequences. Analysis in DIVERGE was conducted to reveal sites of divergent evolution between groups. Three comparisons were conducted; the first compared chloroplast and mitochondrial ClpB sequences in plants. The second comparison identified sites of divergent evolution between ClpC and ClpD sequences in all plant taxa. The final comparison was between fungal lineages, with one group consisting of the ascomycetes and basidiomycetes, and the other fungal group containing all other fungal lineages including the glomeromycetes and zygomycetes.

**Table 1.** DIVERGE analysis of functional changes in gene duplications between chloroplast (CP) and mitochondrial (MT) ClpB sequences. denoted by an asterisk (*).

	Position in our alignment	Site-specific profile: posterior probability	Amino acid(s) Chloroplast	*Arabidopsis thaliana* position number CHLOROPLAST	Amino acid(s) Mitochondria	*Arabidopsis thaliana* position number MITOCHONDRIA	Amino acids(s) Outgroup	*Arabidopsis thaliana* domain	Identified by
D-2	283	1.928336	S, Q, T (Q)	230	E (E)	229	E, Q, A, D, H, T	NBD1	TypeII
D-1	284	1.019195	A, S (A)	231	A, P, T (P)	230	S, T, A	NBD1	TypeII
D-1	300	1.466148	I (I)	246	I, L, V (L)	245	I, V, G	NBD1	Gu2001
D-1	308	0.736892, 1.498342	E (E)	254	E, D (D)	253	E, D	NBD1	Gu99, Gu2001
D-1	345	0.795432, 1.439881	T, G (T)	289	T, M, A, Q (M)	288	R, N, V, T, M, A, G	NBD1	Gu99, Gu2001
D-1	377	0.74898, 1.353602	D (D)	317	K, N, D, T (T)	316	D, S, N	NBD1	Gu99, Gu2001
	387	85.756, 1.135971,	P (P)	327	A, P (P)	326	P, A, K	NBD1	Gu2001, TypeII
D-3*	399	1.506542	D, G, A (D)	339	P, A, G, D, T, V (V)	338	K, D, G, E, A	NBD1	Gu2001
	407	0.715052	V, I (I)	347	V, I A (I)	346	V, I	NBD1	Gu99
	472	0.705567	T (T)	410	T, I (T)	409	V, A, E, T, Q, I, L, D	MD	Gu99
	473	1.849489	A, V (A)	411	A, E (E)	410	E, D, S, A, I, Q, G, V, H	MD	TypeII
	485	0.778969, 0.728913	D, N (D)	416	D (D)	415	D, E	MD	Gu99, TypeII
D-1*	524	0.802913, 1.47656	L, A (L)	450	S, A, M, T, E (T)	449	E, N, D, K, Q, I, V, T, S, A	MD	Gu99, Gu2001
	528	0.940064	R, K (K)	454	K (K)	453	Q, K, E, R, T, G, A	MD	Gu99
D-2*	530	0.973914, 2.84607	K, E, N, R, A, S (A)	456	K (K)	455	Q, R, D, S, A, V, K	MD	Gu99, Gu2001
D-1	533	0.926953, 1.434448	T, N (T)	459	Q, T, S, A, N (N)	458	S, N, M, E, T, L, Q	MD	Gu99, Gu2001
D-1*	538	0.919075, 1.037109	H, R, G (H)	464	M, Q, Y, H, E, K, S, R (K)	463	S, G, A, K, R, T, H	MD	Gu99, Gu2001
D-1	555	0.722169	Q (Q)	473	N, Q, K, G, R (R)	472	N, S, Q, R	MD	Gu99
D-1	557	0.783331, 1.114513	I, L (I)	475	C, I, V, F, L (F)	474	L,V, I, A	MD	Gu99, Gu2001
*	571	1.448217, 1.03127	Q, R (Q)	488	Q, A, S (S)	487	K, Q, R, N	MD	Gu2001, TypeII
D-2	605	0.993931	A, V, T, D, S, E (E)	515	E (E)	514	E, A, D, Q, K, N, V	MD	Gu99
D-3*	642	0.76553	G, A, Q, E (G)	533	A, D (D)	532	E, A, S, D, P	MD and NBD2	TypeII
D-1*	659	1.019195	K (K)	550	R, N, S (N)	549	R, K	NBD2	TypeII
	680	0.847643, 1.48118	V (V)	571	I, V (I)	570	I, V, A	NBD2	Gu99, Gu2001
D-3	694	8.822495	Q (Q)	585	R (R)	584	R, Q	NBD2	TypeII
	731	0.94228	L, M (M)	622	L (L)	621	L, M	NBD2	Gu99
D-3	736	0.761281	E, D (E)	627	K, H, N (N)	626	E, D, K, T	NBD2	TypeII
	782	0.765749, 0.747425	A, S (S)	672	S (S)	671	S, A	NBD2	Gu99, TypeII
D-1*	794	2.365776	S, G (G)	684	G, A, T, Q, H, P (P)	683	L, P, G, A, V, L, S	NBD2	Gu2001
D-1	852	1.028946	R (R)	741	T, R, Q (Q)	740	Q, R, M, I, A	NBD2	TypeII
D-1	854	1.448642	M (M)	743	M, T, I, V (V)	742	L, M, T, E	NBD2	Gu2001
D-1	856	1.434919	A (A)	745	V, I, M, L (L)	744	S, A, V, E, H	NBD2	Gu2001
D-3	880	0.711338	R, K (R)	769	V, S, T (S)	768	R, K, Q, H	NBD2	TypeII
D-2*	915	0.700694	S, G, A, E (A)	799	D, E (E)	798	A, E, K, S	NBD2	Gu99

For the amino acid columns, the letters in the columns represent the amino acid(s) that are located at that position in our alignment, the letter in PARENTHESES represents the residue in the *Arabidopsis thaliana* protein sequence. Posterior probabilities are listed in the same order as “Identified by”, respectively. I.e. “Gu99, G2001” then the p.p. for Gu99 is listed first, then Gu2001. Gu99 – cutoff value of > 0.7; Gu2001- cutoff value of > 1; TypeII – cutoff value of > 0.7. D-1 indicates the sequence was conserved in CP, divergent in MT. D-2 indicates the sequence was conserved in MT, divergent in CP. D-3 indicates divergence between groups. Sites that were identified as significant by other authors are.

**Table 2.** DIVERGE analysis of functional changes in gene duplications between ClpC and ClpD.

	Position in our alignment	Site-specific profile: posterior probability	Amino acid(s) ClpC	*Arabidopsis thaliana* position number ClpC1/ClpC2	Amino acid(s) ClpD	*Arabidopsis thaliana* position number ClpD	*Arabidopsis thaliana* domain	Identified by
D-4	159	1.009727	L, Q (Q)	137	E, P, S, V, I, Q, A (A)	139	ClpN	TypeII
D-4	166	0.905488, 1.482349	L, V, I S (V)	142	R, P, T, D, E, K, Q (K)	145	ClpN	Gu2001, TypeII
D-4*	198	0.81676, 0.878905	-, G, T (G)	153	V, S, R, T, G, A, P (A)	172	ClpN	Gu99, Gu2001
D-4	210	1.173004	R, K, (K)	160/161	Q, S, G, K, E, R, A (A)	181	ClpN	TypeII
D-4	211	1.451017	T, M (M)	161/162	V, T, N, K, P, A, E (K)	182	ClpN	TypeII
D-4	213	1.751453	T (T)	163/164	T, M, A, P, V (V)	184	ClpN	TypeII
D-4	215	0.904156, 0.897083	E, D, (E)	165/166	E, S, D, K, A, N, H, V (E)	186	ClpN	Gu99, Gu2001
	219	1.427909	V, S, T (T)	169/170	V, I, K, L (V)	190	ClpN	TypeII
	220	0.949298, 3.84298	N, D (N)	170/171	D (D)	191	ClpN	Gu2001, TypeII
D-4	223	1.502897	E, Q, K (K)	173/174	A, D, E, Q, L, M, T, V (A)	194	ClpN	TypeII
	226	1.131362	M, I, T, N, A, S, E (E)	176/177	R, A, S, G, D (S)	197	ClpN/AAA	TypeII
D-5	239	0.949298, 3.84298	E, Q (Q)	189/190	E (E)	210	AAA	Gu2001, TypeII
D-4	248	0.80972	G, A (G/A)	198/199	V, G, A, C (C)	219	AAA	Gu2001
	256	12.42123	V, C (C)	206/207	C, V, I (I)	227	AAA	TypeII
*	268	0.821547	I, V (I)	218/219	V, I (I)	239	AAA	Gu2001
D-4	274	1.159889	Q, S (Q)	224/225	Q, T, M, A, I, N, L (I)	246	AAA	TypeII
D-5	282	0.953671	P, R (P)	232/233	P (P)	253	AAA	Gu2001
D-4*	284	4.189404	I, T (T)	234/235	R, M, A, S, F, Y (F)	255	AAA	TypeII
*	285	0.814755, 0.997027	L, I, S (I)	235/236	I, L (L)	256	AAA	Gu99, Gu2001
D-5	290	0.800732, 0.99736	K, R, N T (K/T)	239/240	R (R)	260	AAA	Gu99, Gu2001
D-4*	303	4.154268	T (T)	252/253	T, M, A (A)	273	AAA	TypeII
D-4*	305	12.42123	Y (Y)	254/255	Y, F, E (E)	275	AAA	TypeII
D-4	311	1.020801	E (E)	260/261	E, S, T, A (A)	281	AAA	TypeII
D-4	314	4.16008	K (K)	263/264	K, Q, E, T, N (T)	284	AAA	TypeII
D-4	315	0.726795, 0.829444	K (K)	264/265	S, A, N, K, T (A)	285	AAA	Gu99, Gu2001
	327	0.962812, 0.997067, 1.708534	G, E, D (D)	273/274	G, P, T, N (G)	294	AAA	Gu99, Gu2001, TypeII
D-4	342	2.385296	A (A)	288/289	S, A (S)	309	AAA	TypeII
D-4*	344	1.252882	A (A)	290/291	A, S, G, T, I, V (T)	311	AAA	TypeII
D-4	346	1.236311	E (E)	292/293	D, E, G, N, A, S, K (G)	313	AAA	TypeII
D-4	350	1.029821	G (G)	293/294	G, A, S, T (S)	314	AAA	TypeII
D-4	351	2.597416	A (A)	294/295	A, G, S (G)	315	AAA	TypeII
D-4	379	1.881437	Y (Y)	321/322	Y, H, F (F)	342	AAA	TypeII
D-6*	387	3.971734	A, P (P)	329/330	K, A, G (K)	350	AAA	TypeII
D-4*	390	3.971734	E, (E)	332/333	E, A (A)	353	AAA/MD	TypeII
D-4	399	0.745501	G, P (P)	341/342	E, K, N, D, I, L, H, A (N)	362	MD	Gu99
	402	0.91048	T, S (T)	344/345	S, T (S)	365	MD	Gu2001
D-4	403	1.737264	V, I (V)	345/346	V, I, P, Q, K, E (E)	366	MD	TypeII
	404	0.984168	D, E (D/E)	346/347	D, A, E, V (E)	367	MD	Gu2001
	406	0.903756, 2.522875	T, A (T/A)	348/349	T, A, S (A)	369	MD	Gu2001, TypeII
	407	0.777993	I, T, V (I)	349/350	L, V, I, A (V)	370	MD	Gu2001
D-6	411	0.880696, 0.808178	K, Y, R, H, Q (K/Q)	353/354	R, E, T, Q, L, M (L)	374	MD	Gu99, Gu2001
D-4	422	0.800677	R, K (K)	364/365	G, R, K, N, H (N)	385	MD	Gu99
	423	12.42123	V, L (L)	365/366	V, C (C)	386	MD	TypeII
D-5	426	0.866218, 0.824349	S, T, A, L, D (T)	368/369	S, T (T)	389	MD	Gu99, Gu2001
D-4	427	0.766494	D (D)	369/370	D, P, E, Q, S, T, L F, M (M)	390	MD	Gu99
	428	0.994291, 0.998516	A, E, T, V, D (E)	370/371	A, E, F, G, (E)	391	MD	Gu99, Gu2001
	429	0.712598, 0.975941	A, S (S/A)	371/372	S, A, G (A)	392	MD	Gu99, Gu2001
	432	0.76348	A, S (A)	374/375	S, A, T (A)	395	MD	Gu2001
	436	0.821547	L, F (L)	378/379	L (L)	399	N/A	Gu2001
	439	3.971734	Q, R (Q)	381/382	R (R)	402	MD	TypeII
	811	0.814755	D, N (D)	648/649	N, D (D)	669	AAA2	Gu2001
D-4	818	4.154268	D (D)	655/656	E, C, S, D (S)	676	AAA2	TypeII
	832	0.821547	S, A (S)	669/670	A, S (S)	690	AAA2	Gu2001
D-4	833	0.801291	K, S (S)	670/671	K, A, Q, R, T, S L (L)	691	AAA2	Gu99
D-4	848	1.047427	R (R)	684/685	R, A, T, K, N, Q, G, S, V (G)	705	AAA2/N/A	TypeII
	856	0.821547	E, D (E)	692/693	E (E)	713	N/A	Gu2001
	857	0.906616	L, M (L)	693/694	L, M, V (L)	714	N/A	Gu2001
D-6	879	0.737531, 2.405456	T, S, N (T)	715/716	Q, G, E, S, R (E)	736	N/A	Gu2001, TypeII
D-4	882	3.971734	E (E)	718/719	E, N, V, D, Q (Q)	739	N/A	TypeII

For this analysis, all non-plant sequences were removed, and green algae was used as the outgroup. For the amino acid columns, the letters in the columns represent the amino acid(s) that are located at that position in our alignment, the letter in PARENTHESES represents the residue in the *Arabidopsis thaliana* protein sequence. Posterior probabilities are listed in the same order as “Identified by”, respectively. I.e. “Gu99, G2001” then the p.p. for Gu99 is listed first, then Gu2001. Gu99 – cutoff value of > 0.7; Gu2001- cutoff value of > 1; TypeII – cutoff value of > 0.7. D-4 indicates the residues were conserved in ClpCs and divergent in ClpD. D-5 indicates residues that were conserved in ClpD but divergent in the ClpCs. D-6 indicates that the residues were variable in both species but non-overlapping residues were found in each lineage. Sites that were identified as significant by other authors are denoted by an asterisk (*).

**Table 3.** DIVERGE analysis of functional changes in gene duplications between fungal lineages.

	Amino acid position	Gu99 value	Outgroup	Asco/Basidio	Glomero/Zygo	Saccharomyces HSP104 (JGI)	Saccharomyces HSP104 (NCBI)	Domain (NCBI)	Domain (Desantis Shorter 2012)
D-7	210	0.77698	V	V or T or A	V	L	T, 196	AAA	NBD1
D-7*	279	0.537842	L	L or F or V or I	L	F	F, 265	AAA	NBD1
D-7	306	0.727759	A	Q or N or D or G or A	A	N	N, 292	AAA	NBD1
D-7	308	0.816126	R or S or K	V or K or D or S	K	K	K, 294	AAA	NBD1
D-7	361	0.537842	S or G	P or A or N or K	G	A	A, 341	AAA	NBD1
D-7*	444	0.537842	L	H or Q or I or R	L	Q	Q, 422		MD
D-7	447	0.549232	E	E or Q or D or R	E	Q	Q, 425		MD
D-8*	454	0.62769	E or Q or K	E	A or S or G or E	E	R, 433	Domain of unknown function	MD
D-8	553	0.682409	S or T or Q	E	V or Q or T or E or K	E	E, 521		?
D-8	819	0.83445	A or N	N	R or T or A or H or D or N	N	N, 773	AAA	NBD2

For the amino acid columns, the letters in the columns represent the amino acid(s) that are located at that position in our alignment. Posterior probabilities are listed in the same order as “Identified by”, respectively. I.e. “Gu99, G2001” then the p.p. for Gu99 is listed first, then Gu2001. Gu99 – cutoff value of > 0.7; Gu2001- cutoff value of > 1; TypeII – cutoff value of > 0.7. D-7 indicates that residues are divergent in the ascomycetes and basidiomycetes and conserved in the lineage including the Zygomycetes. D-8 indicates that the residues are conserved in ascomycetes and basidiomycetes and divergent in the other lineage of fungal Hsp104s. Sites that were identified as significant by other authors are denoted by an asterisk (*).

## Results

### Taxon sampling

The final dataset used for this analysis is the largest phylogenetic analysis of ClpB/HSP100 protein family known to date and includes over 240 protein sequences from complete genomes of 69 taxa spanning the tree of life. For a complete list of species and protein accession numbers see supplemental data Tables S1 and S2.

### AlphaFold structure prediction

AlphaFold predicted structure of *Arabidopsis thaliana* ClpB1 (Uniprot accession# P42730) is shown in Figure 1C.^35,73^

### Phylogenetic methods and confidence assessment

**Figure 2.**
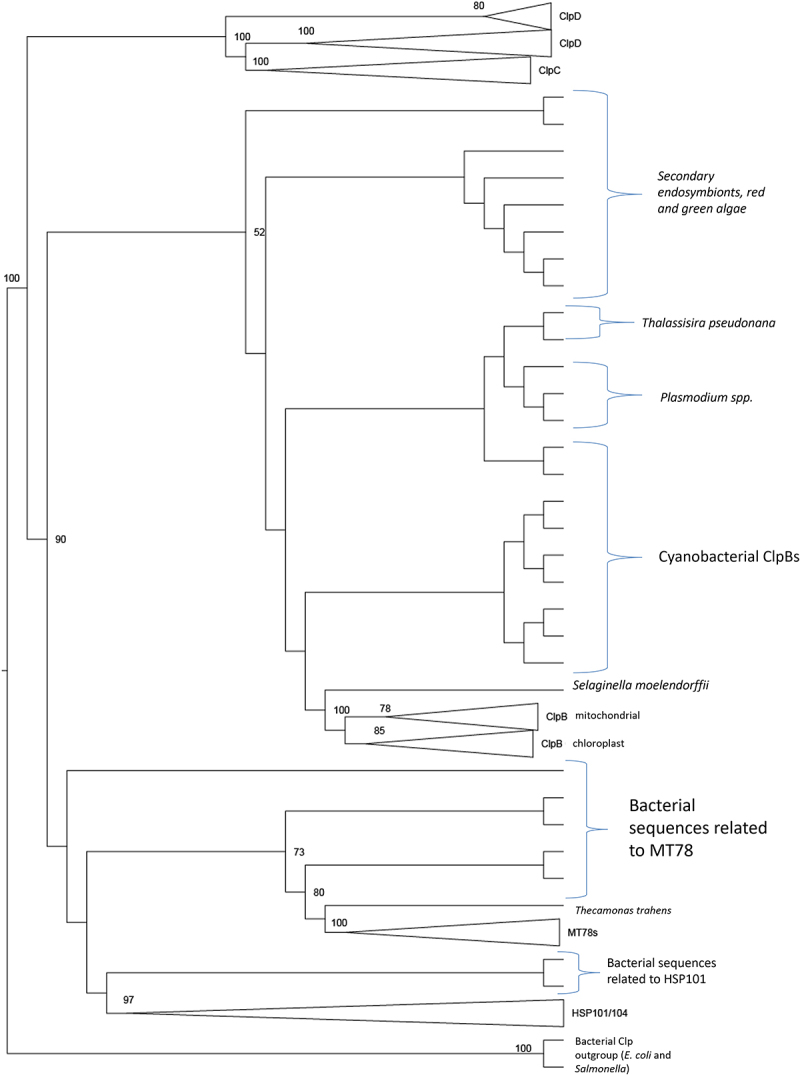
Annotated tree showing relationships between lineages. Phylogenetic relationships among Hsp100 paralogs across bacteria, plastid-bearing eukaryotes, and related lineages. Tree was constructed by collapsing nodes from the complete maximum-likelihood phylogenetic tree of all sequences. Branch lengths are not scaled to rate of evolutionary change. Numbers at nodes indicate bootstrap support.

**Figure 3.**
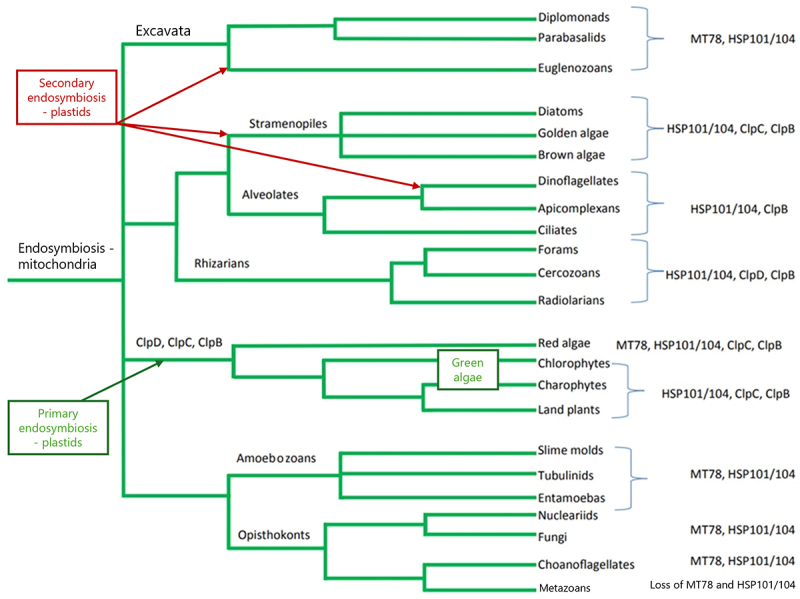
Annotated tree showing gains and losses of HSPs. A phylogenetic tree constructed from complete phylogenetic analysis of all sequences, modified to show broad relationships in the tree of life, with the presence/absence of Clp lineages mapped to each phylogenetic group, as per our analysis. Tree was reconstructed based from Eukaryotic phylogeny in Figure 28.3 in Reece & Campbell^83^ Branch lengths are not scaled to rate of evolutionary change.

The phylogenetic tree analyzing all of the amino acid sequences can be seen in Supplemental Figures 1 and 2. From this larger tree, a simplified gene tree showing the relationships between the five major lineages of ClpB/HSP100 proteins that were detected in this analysis (Figure 2). The distribution of these HSP100 lineages across major organismal groups is indicated in Figure 3. The lineage that contains fungi and animals contains two HSP100 homologues, which we call MT78 (for its mitochondrial origin), and HSP100. Choanoflagellates, the earliest diverging group within the animals, have both of these copies.^10^ Metazoans, however, have lost both copies. Plants, on the other hand, lack MT78s and this implies a secondary loss as they retain cytosolic HSP100s. Plants also have three other lineages of HSP100s which also have homologues in cyanobacteria: ClpDs, ClpCs, and a plant-specific ClpB lineage.

The MT78 lineage is united by a bootstrap of over 90% and thus we concluded that all these sequences share an evolutionary history (Figure 7). An MT78 homolog is found within all fungal genomes examined and form a well-supported clade (Figure 7). These sequences are encoded in the nucleus and the proteins are known to function in the mitochondria in *S. cerevisiae*.^5^ Ancestral to the fungal MT78 sequences are homologs from Choanoflagellates (i.e., *Monosiga sp*. and *Salpingoeca sp*.) and other basal eukaryotes (i.e., *Capsaspora sp*. and *Spaeroforma sp*.) (Figure 7). Ancestral to these sequences in this tree is a polytomy that includes sequences from amoebozoans (i.e., *Dictyostelium sp.)*, alveolates (i.e., *Emiliania sp*.), Excavata (i.e., *Naegleria sp., Trypanosoma spp.*, and *Leishmania*), red algae (i.e., *Cyanidioschyzon sp*.), and Rhizarians (i.e., *Bigelowiella sp*.) (Figure 7).

**Figure 6.**
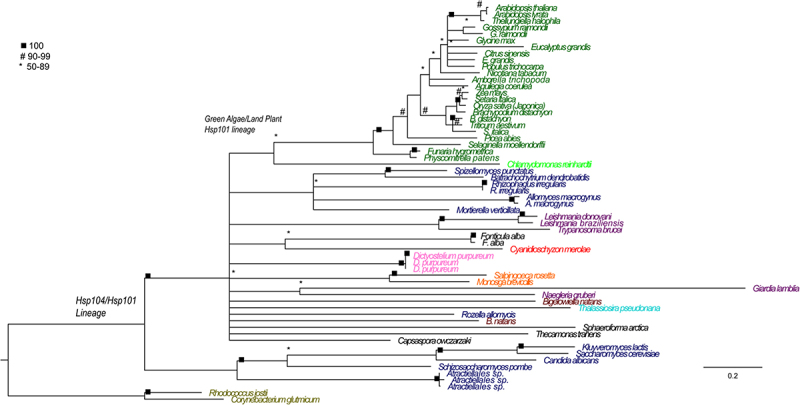
HSP101/104 Maximum likelihood phylogenetic tree constructed using RAxML. 5000 bootstrap replicates were used to determine support. Maximum likelihood tree with best log likelihood score: Protein aligned by OPAL and run with 250 bootstrap replicates in RAxML with rapid bootstrapping under a LG amino acid matrix with categorical gamma and empirical amino acid frequencies used. Support values are bootstrap support (0–100%)

**Figure 7.**
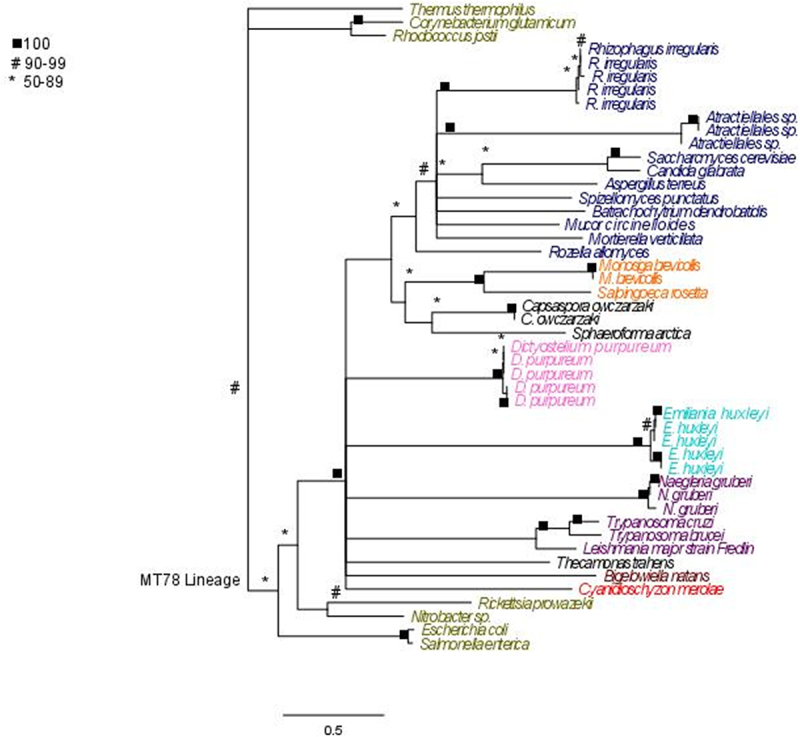
MT78 maximum likelihood phylogenetic tree constructed using RAxML. 5000 bootstrap replicates were used to determine support. Maximum likelihood tree with best log likelihood score: protein aligned by OPAL and run with 250 bootstrap replicates in RAxML with rapid bootstrapping under a LG amino acid matrix with categorical gamma and empirical amino acid frequencies used. Support values are bootstrap support (0–100%).

Of great interest is the evolutionary origins and relationships of the Hsp100/104 homologs. Homologues from diverse organismal origins form a well-supported (100% bootstrap) Hsp101 clade which includes the well-studied fungal Hsp104 homologues. The land plant Hsp101s are also united in a highly supported clade (Figure 6). All land plants examined have an Hsp101 homolog. The branching patterns in this part of the tree reflect organismal relationships indicating a stable pattern of evolution. The Hsp101 homolog of *C. reinhardtii* (a green alga) is found at the base of the land plants. Outside the Green Algal and Land Plant Hsp101 clade, however, is a poorly resolved polytomy. This polytomy includes sequences from fungi (i.e., *Spizellomyces sp., Batrachochytrium sp., Rhizophagus sp., Allomycyes sp.*, and *Mortierella*), Excavata (i.e. *Leishmani sp., Trypanosoma sp*.), red algae (i.e., *Cyanidioschyzon sp*.), Amoebozoa (i.e., *Dictyostelium sp.)*, choanoflagellates (i.e., *Monosiga sp*. and *Salpingoeca sp*.), rhizarians (i.e., *Bigelowiella sp*.), and alveolates (i.e., *Thalassiosira sp*.). The Hsp104 sequences from ascomycetes (including Hsp104 from *S. cervsiae*) and basidiomycetes (including *Atractiellales sp*.) form a well-supported (100% bootstrap) lineage. There is another well-supported lineage of fungal Hsp104s (Figure 6) that includes sequences from Mucoromycota, Glomeromycota and Chytridiomycota. To more fully understand the evolution of fungal phyla we examined these sequences with the program DIVERGE^71^ (Table 3).

**Figure 5.**
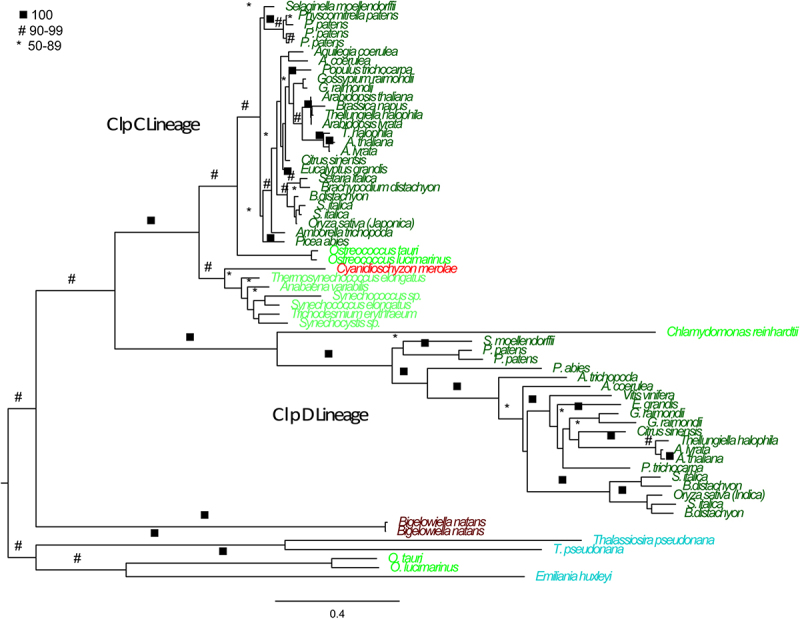
ClpC and ClpD maximum likelihood phylogenetic tree constructed using RAxML. 5000 bootstrap replicates were used to determine support. Maximum likelihood tree with best log likelihood score: protein aligned by OPAL and run with 250 bootstrap replicates in RAxML with rapid bootstrapping under a LG amino acid matrix with categorical gamma and empirical amino acid frequencies used. Support values are bootstrap support (0–100%).

Another lineage of ClpBs that were previously known to be present in plants is the ClpC and ClpD lineage^8^ and is shown in Figure 5. Based on the support for the deep branches within the larger Clp/HSP100 tree, it is clear that the ClpC and ClpD lineages are related to each other and share an evolutionary history. The embryophyte ClpCs are grouped together in a highly supported clade. The phylogenetic relationships at the base of the plant clade do not completely reflect organismal relationships; however, orthologous relationships are clear within the angiosperms (Figure 5). The ClpC lineage also includes sequences from green algae (i.e., *Ostreococcus spp*.), red algae (i.e., *Cyanidioschyzon sp*.), and cyanobacteria (i.e., *Anabaena sp., Trichodesmium sp., Synechococcus spp., Synecocystis sp.*, and *Thermosynechococcus sp*.) (Figure 5). The clade containing ClpD is also well supported and includes one sequence from the green alga *C. reinhardtii* as well as representatives from all the land plants examined (Figure 5). At the base of the ClpC and ClpD lineage we find a number of sequences from the green alga *Ostreococcus* as well as sequences from the Rhizarian *Bigelowiella natans*, the alveolates *Thalassiosira spp*. and *Emiliania spp*. (Figure 5).

**Figure 4.**
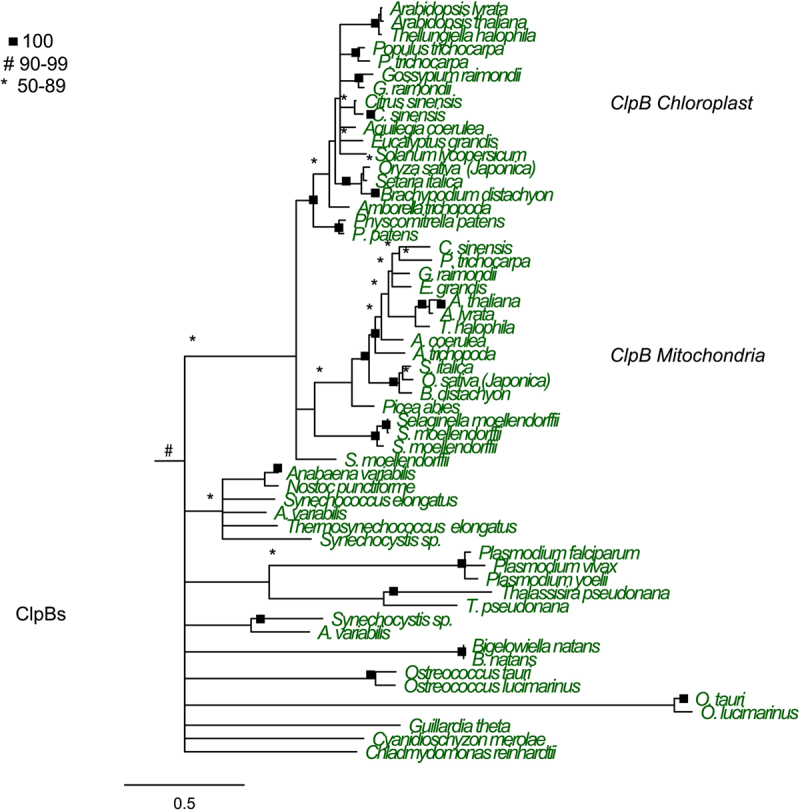
ClpB maximum likelihood phylogenetic tree constructed using RAxML. 5000 bootstrap replicates were used to determine support. Maximum likelihood tree with best log likelihood score: protein aligned by OPAL and run with 250 bootstrap replicates in RAxML with rapid bootstrapping under a LG amino acid matrix with categorical gamma and empirical amino acid frequencies used. Support values are bootstrap support (0–100%).

Phylogenetic analysis of the ClpB lineage within plants shows the expected ClpB chloroplast (ClpB3) and ClpB mitochondrial (ClpB4) lineages predicted by U. Lee and colleagues^8^ and can be seen in Figure 4. The ClpB3 chloroplast group is found in all the plant genomes examined and has high bootstrap support (100). The moss *P. patens* has two ClpB chloroplast homologs but no mitochondrial homolog (Figure 4). The ClpB4 homologs are found in all vascular plants, indicating a single endosymbiotic event that resulted in the establishment of mitochondria in the tracheophytes, prior to the endosymbiotic event that resulted in chloroplasts.^74^ While the angiosperm ClpB4 lineage is highly supported (100), the gymnosperm and *Selaginella* ClpB4 branches have less support (50–89). At the base of the ClpB3 and ClpB4 tree there are some poorly resolved branches that include homologs from cyanobacteria, red and green algae, alveolates and some Rhizarians (Figure 4). This indicates multiple endosymbiotic origins of chloroplasts within these lineages, and secondary and tertiary endosymbiotic events in some lineages.^74^ All of the ClpB sequences are united with a bootstrap support of 90% indicating that they are all members of the same ClpB lineage that share an evolutionary history.

### Analysis of sites undergoing divergent evolution using DIVERGE

The DIVERGE analysis identified 33 key residues that significantly diverge between the chloroplast and mitochondrial ClpB sequences (Table 1). Many of these sites were found in the nucleotide binding regions, but about one-third are of particular interest because they are found within the middle region of the protein located in between the two nucleotide-binding domains. Fifty-nine sequences were identified that differ significantly between the ClpC and ClpD lineages (Table 2). Approximately one-third of these sequences occur in one of the AAA domains of the protein, but the rest are found in portions of the protein without a known function. Finally, 10 sites were found to be significantly divergent in the fungal HSP101/104 lineage, when fungal phylogenetic groups were compared to one another (Table 3). Key residues for that may be important for further study of the function of these proteins have been identified.

## Discussion

Phylogenetic analysis of the plant lineages of HSP100 reveals that land plants obtained one copy of HSP100/ClpB from an ancestor to all eukaryotes, perhaps when obtaining a mitochondrion. This copy resulted in present-day plant HSP100, and subsequently plants lost their copy of MT78. Three additional ClpB copies were obtained by plants from a cyanobacterial ancestor that was the endosymbiotic precursor to the chloroplast, which transferred three separate genes to the plant host. These evolved into ClpB, ClpC, and ClpD in present-day plants. This suggests that during the early endosymbiotic event that produced plastids from cyanobacteria, these three separate genes were transferred to the plant host. Extant cyanobacteria have all three (ClpB, ClpC, and HSP104) of these genes, which supports this hypothesis.^74^ This results in the pattern we see in present-day land plants: based on the Maximum likelihood tree with the best log likelihood score, there are four well-supported (bootstrap 100%), monophyletic plant clades of HSP100. The homologues found in this analysis correspond to the *Arabidopsis thaliana* HSP101 cytosolic nuclear, ClpB mitochondrial and ClpB chloroplast (together in the same clade), ClpD, and ClpC1 and ClpC2 (together in the same clade) that are described in Lee et al. 2007.^8^ As in Lee et al. 2007’s treatment, ClpD and the ClpC clades are sister taxa and are well supported (96% bootstrap support).^8^ These are rooted with good support (96% bootstrap) with bacterial outgroups. While the plant clade itself is well supported (100% bootstrap), the ClpB mitochondrial and ClpB chloroplast group is poorly supported as it is nested within the cyanobacterial and early-diverging eukaryote (Rhizaria) lineages (highest bootstrap support is 68%). For the cytosolic nuclear HSP101, the plant clade is well supported (100% bootstrap) but is poorly supported within cyanobacterial, basal eukaryote, and the Glomeromycota fungi (highest bootstrap support of 83%). The fungal sequences, however, form a well-supported clade (100% bootstrap) with approximately half of the fungi lineages, which we will call the fungi HSP104s. This group is well supported (90% bootstrap) to be sister to another large clade, denoted mitochondrial 78s (MT78s) as it corresponds to Hsp78 described by Erives and Fassler.^10^ The MT78 clade is well supported (100%) to be monophyletic with two lineages: a well-supported lineage (96% bootstrap) containing the fungal proteins (excluding Glomeromycota) and an unsupported lineage with basal eukaryote proteins. The MT78 fungal proteins seem to have no known homologue in the plant lineages, and this is the first study that has incorporated a wide variety of species in an analysis of MT78s, which were previously described only in fungi.^10^

Our phylogenetic analysis revealed five major lineages of ClpB/HSP100 proteins (Figure 2). The earliest evolving lineages we have denoted as MT78 (for its mitochondrial origin), and the HSP101/104 clade. Both of these copies are found in all the groups of eukaryotes studied, which suggests that these were present in a eukaryotic ancestor when eukaryotes first evolved. Choanoflagellates, the earliest diverging group within the animals, have both of these copies, as do fungi.^10^ Eukaryotes retain their copy of HSP100, but MT78 has been lost multiple times in eukaryotic evolution (i.e., in Stramenopiles, Alveolates, Rhizarians, and in Chloropytes including algae and land plants (See Figure 3)). Metazoans, however, have lost both MT78 and HSP100 copies, which is interesting to note because whenever they are found, they are essential for thermotolerance.^6,14,36^ This suggests that the fungal copies of these proteins had their origins when eukaryotes diverged. Plants, on the other hand, lack MT78s and this implies a secondary loss since they retain HSP100s.

Another notable discovery is the distribution of HSP100s in early diverging eukaryotic groups. These include the Rhizaria, Alveolates, Stramenopiles and the polyphyletic “Chromist” lineage, which encompasses red and green algae and plant lineages (Figure 3). While it is clear that this diverse group of eukaryotes received MT78 and HSP101/104 from their eukaryotic ancestor, some of these lineages have ClpC, some ClpD, and some a ClpB that is closely related to the plant ClpBs. Notably, none of these groups have all three of these copies, but they all have at least one copy. The origin of the plastids in these eukaryotes is of particular interest because they have experienced a secondary endosymbiotic event with a red alga.^74,75^ The serial endosymbiotic events that occurred in chromist algae have complicated the analysis of gene evolution in these groups.^76^ This may be why some of them will have ClpD, ClpC, or ClpB but none of them have all these Clp proteins. The red algae in this study have MT78, HSP101, ClpC, and plant ClpB.^74^ The secondary round of endosymbiosis may have caused genes to be lost at random producing the distribution we see today (Figure 3). Further analysis of the function of each of these copies may reveal more clues as to why some copies were kept but others were lost.

Regarding the fungal proteins: there are two well-supported fungal homologue lineages of HSP100; 1) one of these lineages is most closely related to the plant cytosolic nuclear HSP100, hence are recommended to be called fungal HSP104s; and 2) there is a separate lineage of fungal proteins that is well supported as monophyletic but has no known homologue to plant HSP100s, denoted as the MT78s. While it is clear that the “fungal HSP104s” are related to, most likely as sister taxa or perhaps even ancestral to, the plant HSP101s, the origin of the other fungal lineage appears to be from other early-diverging eukaryotic lineages, as this is their sister group. It is interesting to note that a Choanoflagellate (early diverging animal lineage) protein is well-supported (96% bootstrap) to be ancestral to this fungal lineage. This lineage may be, in fact, ancestral to the would-be metazoan HSP100s, which were later secondarily lost in all other Metazoans.^10^ This implies that fungi obtained at least one copy of their HSP100s from a basal eukaryotic ancestor, and the metazoans later lost their copy of HSP100.

We used DIVERGE to analyze the evolutionary dynamics of the ClpB3 and ClpB4 lineages in plants to find amino acid residues that might be evolving under divergent selective constraints (Table 1). This analysis identifies residues that are conserved in one lineage (i.e., ClpB3 or ClpB4) and not in the other. It also identified residues that have divergent patterns of conservation in that the two lineages have different types of amino acids conserved. These amino acid substitutions likely reflect evolutionary adaptations to distinct substrate environments, perhaps found within the organelles. These substitutions may affect binding affinity, substrate specifity, or coordination with co-chaperones. The middle domain, where several of these divergent residues occur, is especially important for regulating substrate engagement and hexamer dynamics. As these organelles have distinct stress environments, the evolution of ClpB family members may have been shaped by the changing nature of their substrate repertoires. This highlights the broader role of molecular chaperones as evolvable systems that adapt not only through gene duplication and loss but also through fine-tuned amino acid changes that modulate their interaction with a dynamic and lineage-specific set of client proteins. Interestingly, in plants, such as *Arabidopsis* and rice, it has been found that SNP-level variation in HSP100 confers differential heat tolerance, particularly at amino acid 907 where an insertion has added a glutamic acid.^12,13^ Our analysis identified 34 residues with divergent patterns of conservation, many of which are previously undescribed and require further investigation. Of these 34 residues, 15 were conserved in ClpB3 proteins but are variable in the ClpB4 proteins (**D-1)**; four residues were conserved in the Clp-B-MT proteins and are variable in the ClpB3 proteins (**D-2)**. Five residues were variable in both the ClpB3 and ClpB4 proteins, but the amino acids found at these positions were non-overlapping across these groups (**D-3)**. Several of these sites have been identified as point mutations that disrupt the molecular chaperone functions of HSP104 proteins.^77,78^ Point mutations in the middle domain have been linked to hexamer destabilization, loss of thermotolerance, and decreased ATPase activity.^23^ For example, the A340V mutation was identified by our analysis (Table 1) and has been shown to be a dominant lethal mutant in HSP104^74^. The A531V and A551T mutations identified in this study were also found by our DIVERGE analysis to be significant sites for divergent evolution.^77^ In addition, we have identified sites of divergent evolution at conserved arginine residues R456, R464, R488, and R769 that are important for ATP hydrolysis of Hsp104 proteins.^78^ The L462R mutation is another key residue for the functional HSP104 hexamer which we found to be under divergent evolution in our analysis.^78^

We were interested in the functional divergence of the ClpCs and ClpDs and performed a DIVERGE analysis on the plant ClpCs and ClpDs (Table 2). There were 59 residues with divergent patterns of selection. Of these, 31 were conserved in ClpCs and divergent in ClpD (**D-4)**. There were four residues that were conserved in ClpD but divergent in the ClpCs (**D-5)**. Interestingly, of these four residues, three were found in the AAA domain. Finally, there were three residues that vary in both groups, however non-overlapping residues were found between the groups (**D-6)**. The residue at position 218 was identified as a site of divergent evolution by our analysis and has been previously identified to be a position in a nucleotide-binding domain where mutations result in reduced chaperone activity by other studies.^11,18^ In addition, several sites at residues between position 250 to 256 were also found to be sites of divergent evolution by our analysis and were identified by Rizo et al. as residues important for substrate interactions in the nucleotide-binding domain 2 of ClpB.^79^ One of these sites (residue 251) is in a pore loop motif and aids substrate translocation through the central channel of the chaperone.^80^ Another site also found as a site of divergent evolution in our study at residue 332 was identified as important in oligomerization and nucleotide hydrolysis.^80^

To examine the patterns of evolution of HSP104 within fungal lineages we compared the sequences of different fungal lineages using the program DIVERGE (Table 3). We compared HSP104 sequences from the fungal subkingdom *Dikarya* containing Ascomycota and Basidiomycota with compartmentalized hyphae to the sequences from of Glomeromycota, Chytridiomycota, and Zygomycota, which have no separation in their hyphae and have continuous cytoplasm in their mycelia.^81,82^ We identified 10 residues with divergent evolutionary patterns. Seven of these residues are divergent in the ascomycetes and basidiomycetes and conserved in the lineage including the Zygomycetes (**D-7)**. The other three residues are conserved in ascomycetes and basidiomycetes and divergent in the other lineage of fungal Hsp104s (**D-8)**. Sites of divergent evolution identified by our analysis that have also been determined by other authors to be significant include E279A located in a Walker B motif, which inhibits ATPase activity and nucleotide hydrolysis while leaving a second AAA module still active.^28,80^ Other notable sites include L444A and Q454A located in the middle domain, which did not alter the ClpB function in a bacterial system.^3^ However, in a yeast system, the missense mutation at the same residue (R444M) impaired ATPase activity of HSP104 and negatively impacted thermotolerance, suggesting an interaction between the distal loop in the middle domain and the second nucleotide-binding domain of HSP104.^23^ This site is likely to be significant in our fungal analysis due to the species-specific collaborations of heat shock proteins.^3^

## Conclusions

While many studies have examined the expression and functions of HSP100s^8,15,,31,36,^ ^37^ to date, this is the first and largest phylogenetic analysis of this protein family examining the evolutionary gains and losses of these proteins across the tree of life in the context of functional divergence. Here, we have examined complete genomes of organisms that are representative of key Eukaryotic lineages for the presence of HSP100/ClpB homologs and have used phylogenetic analysis to determine the evolutionary history of this protein family. Five distinct homologues of HSP100/ClpB were identified by our study. Our analysis reveals the evolutionary relationships of this protein family in Eukaryotes and has identified which homologues are present in each major eukaryotic lineage. This sheds light on the endosymbiotic origins of some of these homologues and provides insight into the mystery of metazoan loss in these proteins. Of note, endosymbiotic events have produced an interesting distribution of these proteins across eukaryotes, and further investigation of this protein family in endosymbionts is warranted. In addition, our analyses have identified specific amino acid residues undergoing divergent evolution, indicating that these residues are functionally significant and will be of future interest in structural analyses of these proteins. The widely conserved distribution of these proteins across the tree of life signifies their importance as molecular chaperones. However, due to the pattern of gene duplications and losses, many organisms have found a way to compensate for their loss by the expansion of other copies fulfilling a similar role. Metazoans are likely to have an evolutionarily distinct yet functionally analogous chaperone system that can be utilized in place of their lost HSP100/ClpB proteins. The biological insights presented in this study enhance our understanding of the evolutionary origins and diversification of the HSP100/ClpB protein family revealing how endosymbiotic events and gene duplication shaped the distribution and specialization of these molecular chaperones across eukaryotes. By identifying key lineage-specific gains and losses, and mapping sites of functional divergence, this work contributes to broader efforts to reconstruct the evolutionary history of molecular chaperone systems and offers a framework for future functional studies of HSP100 proteins in both model and non-model organisms.

## Supplementary Material

Supplemental Figure 2.pdf

Supplemental Figure 1.pdf

Supplemental Table 2.docx

Supplemental Table 1.docx
