# Supplementary figures and images for: Analysis of plant gene family heat shock protein 100 (HSP100) and its orthologs in Eukarya reveals sites of divergent evolution and insights into endosymbiotic origins of chloroplasts
Source: Plant Signal Behav. 2025 Jul 20;20(1):2532008. doi: 10.1080/15592324.2025.2532008 (PMC12283024; doi:10.1080/15592324.2025.2532008)

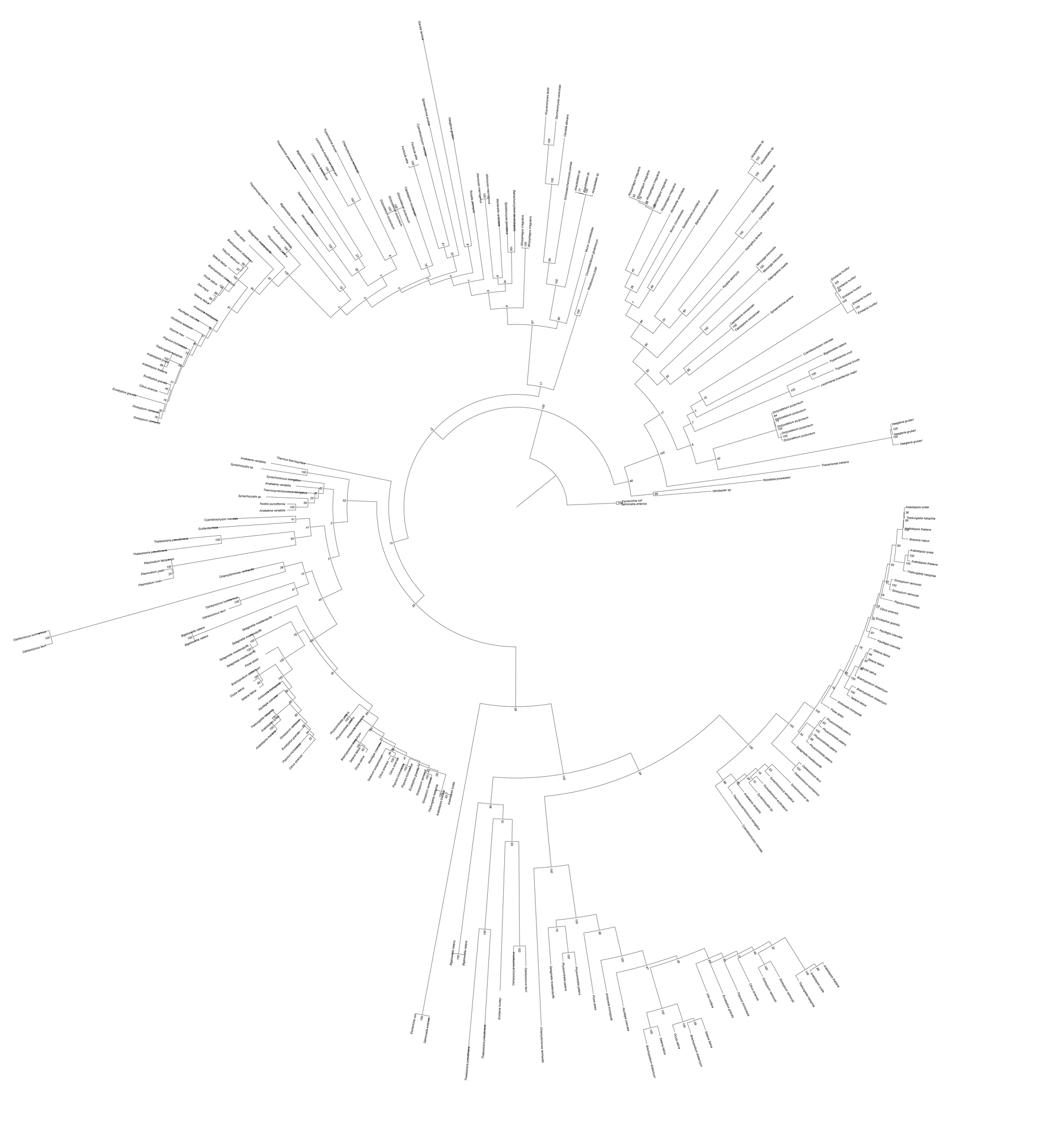

Supplement: Supplemental Figure 1.pdf [file KPSB_A_2532008_SM0097.pdf]
