# Supplementary material for: Analysis of plant gene family heat shock protein 100 (HSP100) and its orthologs in Eukarya reveals sites of divergent evolution and insights into endosymbiotic origins of chloroplasts
Source: Plant Signal Behav. 2025 Jul 20;20(1):2532008. doi: 10.1080/15592324.2025.2532008 (PMC12283024; doi:10.1080/15592324.2025.2532008)
Supplement: Supplemental Table 2.docx [file KPSB_A_2532008_SM0096.docx]

**Supplemental Table 2: List of taxa with sequence accession numbers**

| **Full species name** | **Sequence source** | **Accession #** |
| --- | --- | --- |
| *Allomyces macrogynus* | NCBI  NCBI | KNE63904  KNE67619 |
| *Amborella trichopoda* | NCBI  NCBI  NCBI  NCBI  NCBI  NCBI | XP_006837040  XP_006841133  XP_006844532  XP_006844754  XP_006845519  XP_006857180 |
| *Anabaena variabilis* | NCBI  NCBI  NCBI  NCBI | YP_320409  YP_320661  YP_321429  YP_322848 |
| *Aquilegia coerulea* | Phytozome  Phytozome  Phytozome  Phytozome  Phytozome  Phytozome  Phytozome  Phytozome  Phytozome | Aquca_014_00469.1  Aquca_026_00142.1  Aquca_042_00093.2  Aquca_042_00093.1  Aquca_080_00014.1  Aquca_100_00031.1  Aquca_024_00049.1  Aquca_080_00014.3  Aquca_100_00031.2 |
| *Arabidopsis lyrata* | JGI  JGI  JGI  JGI  JGI  JGI  JGI  JGI  JGI  JGI  JGI  JGI  JGI  JGI  JGI | 108272  488390  355332  325999  57865  495156  330046  330045  355331  173733  68366  320316  485240  495173  481372 |
| *Arabidopsis thaliana* | NCBI  NCBI  NCBI  NCBI  NCBI  NCBI | NP_568746  NP_001190035  NP_565083  NP_565586  NP_568314  NP_568750 |
| *Aspergillus terreus* | NCBI | XP_001210406 |
| *Atractiellales sp.* | JGI  JGI  JGI  JGI  JGI  JGI  JGI  JGI  JGI  JGI  JGI  JGI  JGI  JGI  JGI  JGI  JGI  JGI  JGI | 1311997  1314289  1205599  1216476  1203856  1393710  1313433  1377043  1194754  1206481  1348708  1183648  1184506  1199369  1295602  1307921  1309427  1185135  1307239 |
| *Batrachochytrium dendrobatidis* | JGI  JGI | 31536  8849 |
| *Bigelowiella natans* | JGI  JGI  JGI  JGI  JGI  JGI  JGI  JGI  JGI  JGI  JGI  JGI  JGI | 36657  36914  47555  57897  79706  141716  18894  57526  71933  37002  28563  18796  125406 |
| *Brachypodium distachyon* | Phytozome  Phytozome  Phytozome  Phytozome  Phytozome  Phytozome  Phytozome  Phytozome  Phytozome  Phytozome | Bradi2g19540.1  Bradi2g49660.1  Bradi4g20470.1  Bradi3g44640.1  Bradi3g44340.3  Bradi3g44340.1  Bradi1g16190.1  Bradi3g06107.1  Bradi4g39880.1  Bradi5g08920.1 |
| *Brassica napus* | NCBI | CLPA_BRANA |
| *Candida albicans* | NCBI | XP_716146 |
| *Candida glabrata* | NCBI | XP_449729 |
| *Capsaspora owczarzaki* | NCBI | XP_004348638 |
| *Chlamydomonas reinhardtii* | NCBI  NCBI  NCBI | XP_001698633  XP_001698806  XP_001701777 |
| *Citrus sinensis* | Phytozome  Phytozome  Phytozome  Phytozome  Phytozome  Phytozome | orange1.1g002068m  orange1.1g002623m  orange1.1g046258m  orange1.1g002012m  orange1.1g003088m  orange1.1g003094m |
| *Corynebacterium glutamicum* | NCBI | NP_601973 |
| *Cyanidioschyzon merolae* | NCBI | XP_005538794  XP_005535337  XP_005536403  XP_005538794  NP_849021 |
| *Dictyostelium purpureum* | NCBI | XP_003285601  XP_003289536 |
| *Dictyostelium purpureum* | JGI | 15538  4947  88557  35923  35912  5190 |
| *Emiliania huxleyi* | JGI | 66245  75681  66291  75684  75680  66269  262459  262460  40954  40968  40884  265034  265036  265035  278708  246308  76412  76418  40878  40973  40998  40953  40955  283729  115562 |
| *Escherichia coli* | NCBI | AAA24422  YP_851973 |
| *Eucalyptus grandis* | Phytozome | Eucgr.H00243.1  Eucgr.H00243  Eucgr.K02198  Eucgr.K02198  Eucgr.K02521  Eucgr.B00581  Eucgr.I01982  Eucgr.C02666 |
| *Fonticula alba* | NCBI | XP_009497595.1 |
| *Funaria hygrometrica* | NCBI | ABB73202 |
| *Giardia lamblia ATCC 50803 (Giardia intestinalis ATCC 50803)* | NCBI | XP_001709001 |
| *Glycine max (soybean)* | NCBI | NP_001238122 |
| *Gossypium raimondii* | Phytozome | Gorai.001G053000.1  Gorai.003G100900.1  Gorai.001G077600.1  Gorai.010G118200.1  Gorai.010G118200.2  Gorai.012G050100.1  Gorai.006G011800.1  Gorai.006G273600.1  Gorai.001G054700.1  Gorai.003G116200.2  Gorai.003G116200.1 |
| *Guillardia theta* | JGI | 159075  55776  57813  91333  160401 |
| *Kluyveromyces lactis* | NCBI | XP_455896 |
| *Leishmania braziliensis* | NCBI | XP_001566479 |
| *Leishmania donovani* | NCBI | CAB08073 |
| *Leishmania major strain Friedlin* | NCBI | XP_003721676 |
| *Monosiga brevicollis* | JGI | 28355  21607 |
| *Mortierella verticillata* | NCBI | KFH63991 |
| *Mucor circinelloides* | JGI | 127172  127181  154401  151933  159842  106724  137708  72518  127511  127445  127444  139240 |
| *Naegleria gruberi* | JGI | 321  332  38879  32924  55744  60558  446  437  33729  67914  60646  36796 |
| *Nicotiana tabacum (common tobacco)* | NCBI | AAC83688 |
| *Nitrobacter sp.* | NCBI | WP_009798401 |
| *Nostoc punctiforme* | NCBI | YP_001867738 |
| *Oryza sativa Indica Group (long-grained rice)* | NCBI | AAN78327 |
| *Oryza sativa Japonica Group (Japanese rice)* | NCBI | CLPB3_ORYSJ  CLPC1_ORYSJ  NP_001050410  NP_001056066 |
| *Ostreococcus lucimarinus* | NCBI | XP_001415741  XP_001417871  XP_001418921  XP_001422509 |
| *Ostreococcus tauri* | NCBI | XP_003074131  XP_003079585  XP_003080314  XP_003082910 |
| *Physcomitrella patens* | NCBI | XP_001752142  XP_001756645  XP_001757304  XP_001763692  XP_001766010  XP_001766888  XP_001775725  XP_001779162  XP_001781353 |
| *Picea abies (Norway spruce)* | Congenie.org | MA_763  MA_10427039  MA_8475  MA_8475  MA_229920  MA_178786  MA_31115  MA_105215  MA_8117  MA_596288  MA_214624  MA_9507074g0030  MA_158805g0170   MA_159146g0040 |
| *Plasmodium falciparum* | NCBI | XP_001349358 |
| *Plasmodium vivax* | NCBI | XP_001615005 |
| *Plasmodium yoelii yoelii* | NCBI | XP_725369 |
| *Populus trichocarpa (Populus balsamifera subsp. trichocarpa)* | NCBI | XP_002305376  XP_002308700  XP_002318194  XP_002322299  XP_002324092  XP_006374393 |
| *Rhizophagus irregularis* | JGI | 32602  214957  44616  71519  45529  71517  54417  71518  45965  39575  299904  12285  89346 |
| *Rhodococcus jostii* | NCBI | YP_705452 |
| *Rickettsia prowazekii str. Madrid E* | NCBI | NP_220430 |
| *Rozella allomycis* | JGI | 86  3519  1350 |
| *Saccharomyces cerevisiae* | NCBI | NP_010544  NP_013074 |
| *Salmonella enterica subsp. enterica serovar Typhi str. CT18* | NCBI | NP_455435  NP_457131 |
| *Salpingoeca rosetta* | NCBI | XP_004995761 |
| *Schizosaccharomyces pombe* | NCBI | NP_596503 |
| *Selaginella moellendorffii* | Phytozome | 170696  411118  418380  231598  174539  439163  147913 |
| *Setaria italica* | Phytozome | Si021144m  Si021149m  Si000232m  Si034086m  Si016214m  Si016215m  Si021139m  Si009304m  Si009306m  Si009283m  Si016236m |
| *Solanum lycopersicum (Lycopersicon esculentum)* | NCBI | NP_001234143 |
| *Sphaeroforma arctica* | NCBI | XP_014148202 |
| *Spizellomyces punctatus* | NCBI | XP_016607280 |
| *Synechococcus elongatus* | NCBI | YP_399279  YP_400106 |
| *Synechococcus sp. WH 8102* | NCBI | NP_897031 |
| *Synechocystis sp. PCC 6803* | NCBI | NP_441776  NP_441882  NP_442112 |
| *Thalassiosira pseudonana* | NCBI | XP_002292380  XP_002295000  XP_002286314  XP_002288245  XP_002288746 |
| *Thecamonas trahens* | NCBI | XP_013757397 |
| *Thellungiella halophila* | Phytozome | Thhalv10018095m  Thhalv10000031m  Thhalv10012593m  Thhalv10010105m  Thhalv10012603m  Thhalv10010143m  Thhalv10012591m  Thhalv10012603m |
| *Thermosynechococcus elongatus* | NCBI | NP_681098  NP_682179 |
| *Thermus thermophilus* | NCBI | YP_005092 |
| *Trichodesmium erythraeum* | NCBI | YP_722125 |
| *Triticum aestivum (bread wheat)* | NCBI | AF174433_1 |
| *Trypanosoma brucei brucei* | NCBI | XP_951576  XP_951738 |
| *Trypanosoma cruzi strain CL Brener* | NCBI | XP_817674 |
| *Vitis vinifera (wine grape)* | NCBI | XP_002283802 |
| *Zea mays* | NCBI | NP_001104935 |
