# Supplementary material for: Analysis of plant gene family heat shock protein 100 (HSP100) and its orthologs in Eukarya reveals sites of divergent evolution and insights into endosymbiotic origins of chloroplasts
Source: Plant Signal Behav. 2025 Jul 20;20(1):2532008. doi: 10.1080/15592324.2025.2532008 (PMC12283024; doi:10.1080/15592324.2025.2532008)
Supplement: Supplemental Table 1.docx [file KPSB_A_2532008_SM0095.docx]

**Supplemental Table 1: Complete list of sampled taxa**

| **Full species name** | **Group** |
| --- | --- |
| *Corynebacterium glutamicum* | Bacteria - Actinobacteria |
| *Rhodococcus jostii* | Bacteria - Actinobacteria |
| *Thermus thermophilus* | Bacteria - Deinococcus |
| *Escherichia coli* | Bacteria - Proteobacteria |
| *Nitrobacter sp.* | Bacteria - Proteobacteria |
| *Rickettsia prowazekii* | Bacteria - Proteobacteria |
| *Salmonella enterica subsp. enterica* | Bacteria - Proteobacteria |
| *Plasmodium falciparum* | Basal Eukaryote - Alveolate |
| *Plasmodium vivax* | Basal Eukaryote - Alveolate |
| *Plasmodium yoelii yoelii* | Basal Eukaryote - Alveolate |
| *Dictyostelium purpureum* | Basal Eukaryote - Amoebozoa |
| [*Guillardia theta*](http://genome.jgi.doe.gov/Guith1) | Basal Eukaryote - Cryptophyta |
| *Leishmania braziliensis* | Basal eukaryote - Euglenozoa |
| *Leishmania donovani* | Basal eukaryote - Euglenozoa |
| *Leishmania major* | Basal eukaryote - Euglenozoa |
| *Trypanosoma brucei brucei* | Basal eukaryote - Euglenozoa |
| *Trypanosoma cruzi* | Basal eukaryote - Euglenozoa |
| [*Emiliania huxleyi*](http://genome.jgi.doe.gov/Emihu1) | Basal Eukaryote - Haptophyta |
| *Thalassiosira pseudonana* | Basal Eukaryote - Heterokonta |
| [*Naegleria gruberi*](http://genome.jgi.doe.gov/Naegr1) | Basal Eukaryote - Heterolobosea |
| *Giardia lamblia* | Basal eukaryote - Metamonada |
| [*Bigelowiella natans*](http://genome.jgi.doe.gov/Bigna1) | Basal Eukaryote - Rhizaria |
| [*Monosiga brevicollis*](http://genome.jgi.doe.gov/Monbr1) | Choanoflagellate (basal animal) |
| *Anabaena variabilis* | Cyanobacteria |
| *Nostoc punctiforme* | Cyanobacteria |
| *Synechococcus elongatus* | Cyanobacteria |
| *Synechococcus sp.* | Cyanobacteria |
| *Synechocystis sp.* | Cyanobacteria |
| *Thermosynechococcus elongatus* | Cyanobacteria |
| *Trichodesmium erythraeum* | Cyanobacteria |
| *Aspergillus terreus* | Fungi - Ascomycota |
| *Candida albicans* | Fungi - Ascomycota |
| *Candida glabrata* | Fungi - Ascomycota |
| *Kluyveromyces lactis* | Fungi - Ascomycota |
| *Saccharomyces cerevisiae* | Fungi - Ascomycota |
| *Schizosaccharomyces pombe* | Fungi - Ascomycota |
| [*Atractiellales sp.*](http://genome.jgi.doe.gov/Atrsp2) | Fungi - Basidiomycota |
| [*Batrachochytrium dendrobatidis*](http://genome.jgi.doe.gov/Batde5) | Fungi - Chytridiomycota |
| [*Rozella allomycis*](http://genome.jgi.doe.gov/Rozal1_1) | Fungi - Cryptomycota |
| *Rhizophagus irregularis* | Fungi - Glomeromycota |
| [*Mucor circinelloides*](http://genome.jgi.doe.gov/Mucci2) | Fungi - Mucorormycotina |
| *Chlamydomonas reinhardtii* | Green algae |
| *Ostreococcus lucimarinus* | Green algae |
| *Ostreococcus tauri* | Green algae |
| *Cyanidioschyzon merolae* | Rhodophyta |
| *Aquilegia coerulea* | Viridiplantae |
| [*Arabidopsis lyrata*](http://genome.jgi.doe.gov/Araly1) | Viridiplantae |
| *Arabidopsis thaliana* | Viridiplantae |
| *Brassica napus* | Viridiplantae |
| *Citrus sinensis* | Viridiplantae |
| *Eucalyptus grandis* | Viridiplantae |
| *Glycine max* (soybean) | Viridiplantae |
| *Gossypium raimondii* | Viridiplantae |
| *Nicotiana tabacum* (common tobacco) | Viridiplantae |
| *Populus trichocarpa (Populus balsamifera subsp. trichocarpa)* | Viridiplantae |
| *Solanum lycopersicum (Lycopersicon esculentum)* | Viridiplantae |
| *Thellungiella halophila* | Viridiplantae |
| *Vitis vinifera* (wine grape) | Viridiplantae |
| *Amborella trichopoda* | Viridiplantae (basal Angiosperm) |
| *Funaria hygrometrica* | Viridiplantae (Bryophyte) |
| *Physcomitrella patens* | Viridiplantae (Bryophyte) |
| *Picea abies* (Norway spruce) | Viridiplantae (Gymnosperm) |
| *Selaginella moellendorffii* | Viridiplantae (Lycopod) |
| *Brachypodium distachyon* | Viridiplantae (Monocot) |
| *Oryza sativa Indica Group* (long-grained rice) | Viridiplantae (Monocot) |
| *Oryza sativa Japonica Group* (Japanese rice) | Viridiplantae (Monocot) |
| *Setaria italica* | Viridiplantae (Monocot) |
| *Triticum aestivum* (bread wheat) | Viridiplantae (Monocot) |
| *Zea mays* | Viridiplantae (Monocot) |
